# Unconventional data, unprecedented insights: leveraging non-traditional data during a pandemic

**DOI:** 10.3389/fpubh.2024.1350743

**Published:** 2024-03-07

**Authors:** Kaylin Bolt, Diana Gil-González, Nuria Oliver

**Affiliations:** ^1^Health Sciences Division (Assessment, Policy Development, and Evaluation Unit), Public Health - Seattle & King County, Seattle, WA, United States; ^2^Department of Community Nursing, Preventive Medicine and Public Health and History of Science, University of Alicante, Alicante, Spain; ^3^CIBER Epidemiología y Salud Pública (CIBERESP), Madrid, Spain; ^4^European Laboratory for Learning and Intelligent Systems (ELLIS) Alicante, Alicante, Spain

**Keywords:** non-traditional data, COVID-19, data governance, precision public health, digital health

## Abstract

**Introduction:**

The COVID-19 pandemic prompted new interest in non-traditional data sources to inform response efforts and mitigate knowledge gaps. While non-traditional data offers some advantages over traditional data, it also raises concerns related to biases, representativity, informed consent and security vulnerabilities. This study focuses on three specific types of non-traditional data: mobility, social media, and participatory surveillance platform data. Qualitative results are presented on the successes, challenges, and recommendations of key informants who used these non-traditional data sources during the COVID-19 pandemic in Spain and Italy.

**Methods:**

A qualitative semi-structured methodology was conducted through interviews with experts in artificial intelligence, data science, epidemiology, and/or policy making who utilized non-traditional data in Spain or Italy during the pandemic. Questions focused on barriers and facilitators to data use, as well as opportunities for improving utility and uptake within public health. Interviews were transcribed, coded, and analyzed using the framework analysis method.

**Results:**

Non-traditional data proved valuable in providing rapid results and filling data gaps, especially when traditional data faced delays. Increased data access and innovative collaborative efforts across sectors facilitated its use. Challenges included unreliable access and data quality concerns, particularly the lack of comprehensive demographic and geographic information. To further leverage non-traditional data, participants recommended prioritizing data governance, establishing data brokers, and sustaining multi-institutional collaborations. The value of non-traditional data was perceived as underutilized in public health surveillance, program evaluation and policymaking. Participants saw opportunities to integrate them into public health systems with the necessary investments in data pipelines, infrastructure, and technical capacity.

**Discussion:**

While the utility of non-traditional data was demonstrated during the pandemic, opportunities exist to enhance its impact. Challenges reveal a need for data governance frameworks to guide practices and policies of use. Despite the perceived benefit of collaborations and improved data infrastructure, efforts are needed to strengthen and sustain them beyond the pandemic. Lessons from these findings can guide research institutions, multilateral organizations, governments, and public health authorities in optimizing the use of non-traditional data.

## 1 Introduction

In our increasingly data-driven society, understanding the value and limitations of non-traditional data for public health is crucial. For this article, we define non-traditional data as “*data that is digitally captured, mediated, or observed*” (1). This study focuses specifically on mobility, participatory surveillance and social media data, as described in Table 1.

**Table 1:** Non-traditional data sources included study.

**Area**	**Description (2)**	**Sources included in study**
Mobility	Non-traditional data that describes the location of a device relative to people and landmarks in the physical world or to other devices	*Mobility data* •Telecommunication companies (ex: call detail records data) •Location Intelligence Companies (ex: Software development kit data)
Sentiment	Non-Traditional data that describes how individuals and groups perceive and understand developments related to COVID-19	*Social media data* •Activity (posts, likes, hashtags) on Twitter, Facebook, Instagram, etc.
Health	Non-traditional data that describes, or can be used as a proxy for, the physical health of individuals or populations, especially as it relates to COVID-19 diagnoses	*Participatory surveillance* •Platform-facilitated symptom data

Descriptions sourced from Chafetz et al. (2).

The COVID-19 pandemic was recognized by many as a syndemic due to its multifaceted impact and interaction with other heath conditions and societal environments (3, 4). Acknowledging the role of inequality in adverse health outcomes, this perspective underscores the necessity for a multifaceted public health response to COVID-19. Thus, the complexity and urgency of the pandemic prompted many policymakers and researchers to adopt non-traditional data and AI-based methods for public health surveillance, informatics, and decision making (2, 5). However, and despite promising insights, concerns around inaccuracies, biases, privacy implications, and security flaws related to this type of data still persist.

Over the past decade, non-traditional data sources gained visibility in the public domain through the production of official statistics (6, 7) and measuring progress toward the UN's Sustainable Development Goals (SDG) (8, 9). The rise of non-traditional data has been driven by the availability of massive non-structured data from widespread smartphone adoption and digitization of physical space (10). Big data offers diverse applications in public health, and has gained relevance through digital epidemiology (11), public health informatics, and the precision public health movement (12, 13).

Prior to the pandemic, non-traditional data sources had been applied to public health in various ways. Mobility data has helped model the spread of multiple infectious diseases (14–16). Participatory surveillance has monitored influenza-like illness (ILI) in nearly real-time (17), while mitigating data gaps and reporting lags of traditional surveillance (18) and showcasing significant citizen engagement (19). Moreover, social media sentiment data has been applied to forecast disease outbreaks (20) and assess public opinion on vaccines (21), with growing potential to aid public health campaigns and interventions (22).

The COVID-19 pandemic further expanded the presence of non-traditional data in public health research and data collection, with many calling for their adoption (23, 24). Insights were obtained through smartphone apps (25), wearable sensors (26), and wastewater (27). Additionally, as further explored in this study, social media captured public sentiment (28), participatory surveillance gathered COVID-19 trends and health information (29) and mobility data was used to monitor lockdown compliance and disease spread (30). Despite offering promising insights, concerns regarding privacy, consent, security flaws, and data quality presents challenges to those weighing the immediate utility of non-traditional data against limitations.

The inter-pandemic phase offers an opportunity to assess lessons learned and identify positive changes that should be sustained. This research study aims to identify key takeaways from using non-traditional data during the COVID-19 pandemic in Spain and Italy. To the authors' knowledge, no study has adopted a methodology based on in-depth interviews to explore non-traditional data uses during a pandemic. While existing studies demonstrate the utility of non-traditional data in public health, none have investigated the experiences of stakeholders applying them to research, practice, and policy. Furthermore, there is a lack of qualitative research examining the implementation of non-traditional data in Spain and Italy, the primary focus of this research.

This paper seeks to address these gaps through a systematic analysis of interviews with multidisciplinary key informants on their utilization of non-traditional data in Spain and Italy during the COVID-19 pandemic. The experiences of those who leveraged this type of data during the COVID-19 pandemic can deepen our awareness of what they offer to public health, as well as how to address risks and limitations. The interviews aimed to gather perspectives on successes, challenges, and future opportunities. Examining these use cases provides insights on the value, risks, and potential of non-traditional data for public health and beyond. This research contributes novel insights to the literature and offers relevant considerations for agencies, including health departments, research institutes, corporations, and government entities, contemplating the use of these data sources to address public health challenges.

## 2 Materials and methods

### 2.1 Study setting

The focus of Spain and Italy for this study was driven by the severe impact of COVID-19 in both countries, as well as the similarities in their healthcare models. Practical considerations, including language proficiency of study team and collaborations with pertinent research institutes in each country also played a role in the selection process. The global COVID-19 pandemic, resulting in over 6 million deaths worldwide, has had unprecedented consequences on social, economic and political aspects of life (31). Italy and Spain, the first European Union member states impacted, were initially among the hardest-hit in the world. According to March 2023 estimates, Spain experienced a total of 119,479 COVID-19-related deaths and Italy a total of 188,322 (32). In response to the pandemic, researchers and government officials in both countries initiated endeavors to harness non-traditional data. In Italy, these included participatory surveillance platforms (33), social media data (34), and wastewater-based epidemiology (35), among others. Similarly, non-traditional data was employed in Spain through mobility (36), wastewater (37), and participatory methods (38). Within each country in-depth, semi-structured interviews were conducted with key informants who utilized non-traditional data sources during the COVID-19 pandemic. The goal of these interviews was to gather perspectives on the factors that facilitated utilization, challenges faced, and recommendations for future use.

### 2.2 Participants

Eligible candidates for this study were identified based on their expertise and experience working with non-traditional data during the pandemic. Interviewees worked with one or more of the following types of non-traditional data: mobility, participatory surveillance, and social media. Interviews were also conducted with representatives of biosensor and wastewater surveillance data but were later excluded from analysis due to an insufficient sample size to generate reliable conclusions. Participants had a range of backgrounds, including epidemiology, data science, public policy, and artificial intelligence. They practiced in diverse settings, including universities, research institutes, public health agencies, government offices, and private companies. Criteria for inclusion required participants to be 18 years or older, be comfortable participating in Spanish or English, and have resided in Spain or Italy for most of the period between March 2020 to March 2022. The majority of participants from Spain were based in the Valencian Autonomous Community or Region, while the majority from Italy were in the Piedmont Region. Researchers sought a balanced sample by gender, country, and area of expertise, although achieving this was not always feasible. Recruiting female interviewees for this study proved challenging, particularly in Spain, which authors believe is due to the widespread under-representation of women in technical fields like data science and artificial intelligence (39, 40).

Key informants were recruited using purposive and snowball sampling methods. Initial interviewees were first nominated based on investigators' knowledge and through interviewee affiliations with research institutions, local government offices, or COVID-19 response taskforces. Snowball sampling was employed as needed, with interviewees providing recommendations of additional key informants. Recruitment was concluded when data saturation (41) was reached, with a minimum of eight interviewees. Candidates were contacted via email to explain the study, interview purpose, and invite participation. If no response was received, a follow up email was sent as a final notification.

### 2.3 Collection and treatment of information

All interview participants received a copy of the consent form and information sheet before the interview. At the interview's outset, participants confirmed receipt of these forms and were invited to share any questions or concerns. Consent was provided electronically through a Google Forms document prior to the interview and through verbal agreement at the beginning of the interview.

Interviews were completed between February and June 2023, and took place face-to-face, over virtual video platform, or by telephone, based on the interviewee's preference. Interviews were conducted in Spanish or English, lasting between 0.5 and 2.0 h, with a median duration of 53.5 min. A 12-question topic guide was developed by the three primary investigators and is included in Supplementary material. Question development was informed by an extensive literature review, the collective knowledge of the authors, and insights from experts in public health and non-traditional data. All questions were open-ended, supplemented with optional probes for additional detail. The topic guide covered key areas of interest and was divided into seven sections: an initial question on professional background, utility of non-traditional data, facilitators of use, challenges of use, privacy and equity, impact of use, and recommendations for future use. The primary investigators considered relevance of each question to the overall research goals, clarity of wording, and interview duration when finalizing the guide. Throughout the interview process, if unanticipated but pertinent issues arose they were explored through follow-up questions. Though interviews primarily followed sequential order of the topic guide occasionally this was modified and in rare occasions questions were skipped if the participant had time-constraints.

Audio recordings of interviews were transcribed verbatim using Sonix audio transcription software, then reviewed manually by the lead researcher to verify accuracy. Areas of uncertainty in Spanish were reviewed by native Spanish-speaking co-investigators. Quotes from the transcripts are included to highlight participant perspectives. Some have been condensed or modified for brevity and/or clarity, however any alterations aim to preserve participants' intended meaning within the limitations of space. This study received IRB approval through the Ethics Committee of the University of Alicante (Code: I0135).

### 2.4 Data analysis

We conducted an applied thematic analysis (42), using both inductive and deductive approaches. This methodology has been valuable for public health research and offers a systematic approach to coding and identifying key themes across different informants.

Transcriptions were systematically coded using Dedoose 9.0.82 software program. The initial codebook featured concepts that were predefined based on the topic guide, with subsequent integration of inductive codes that emerged from data content. The codebook was iteratively refined during the initial coding phase, where a subset of transcripts were reviewed. As a result, some codes were merged, disaggregated, or reassigned while new concepts were added before finalization. Questions and feedback from co-investigators on codes or themes were discussed and resolved through mutual agreement.

Four overarching themes were identified that aligned with the topic guide, as well as additional subthemes. The lead researcher applied a framework matrix approach (43) to organize and compare coded data across participants. This approach is useful for comparing and contrasting data, which was of particular importance to this study given the distinct characteristics of interviewees. This matrix structure facilitated easier comparisons by country, type of data expertise, and background of interviewee. This method proved effective in highlighting variations and commonalities across different domains of the data, contributing to a comprehensive understanding of the study's findings. A narrative summary was then written to describe and synthesize these data by theme.

## 3 Results

### 3.1 Participants

A total of 18 participants were interviewed: 12 males and six females, all with experience applying non-traditional data to pandemic-related efforts in Spain or Italy. There was disproportionate representation of interviewees experienced with mobility data, compared to participatory surveillance and social media. Table 2 outlines participant characteristics.

**Table 2:** Participant profiles and demographics (*N* = 18).

**ID**	**Sex**	**Role**	**Workplace**	**Country**
P01	F	Research	Non-profit research institute	Italy
P02	F	Research	Non-profit research institute	Italy
P03	F	Research	Public research institute	Italy
P04	F	Research	Non-profit research institute	Italy
P05	M	Research	Non-profit research institute	Italy
P06	M	Research	Non-profit research institute	Italy
P07	M	Research	Non-profit research institute	Italy
P08	M	Research	Non-profit research institute	Italy
P09	M	Research	Public university	Spain
P10	M	Research	Public university	Spain
P11	M	Research	Public university	Spain
P12	M	Research	Public university	Spain
P13	M	Research	Public university	Spain
P14	M	Research	Public university	Spain
P15	M	Public official	Government agency (statistics)	Spain
P16	M	Artificial intelligence	Telecommunication company	Spain
P17	F	Public official	Government agency (public health)	Spain
P18	F	Public official	Government agency (public policy)	Spain

### 3.2 Key themes and summary of the findings

We identified four main themes, which were subdivided into various subthemes. Primary themes included (1) value of non-traditional data, (2) facilitators of use, (3) challenges of use, and (4) recommendations for future. The subsequent sections in this article describe each key theme separately.

#### 3.2.1 Theme 1: value of non-traditional data

The value of non-traditional data was described through two sub-themes: (1) unique advantages leveraged during the pandemic and (2) the underutilization of their potential, especially within the public sector.

##### 3.2.1.1 Subtheme 1: unique advantages leveraged during pandemic

Participants highlighted unique advantages of non-traditional data during the pandemic, including the ability to address critical data gaps, offer distinct perspective on behavior, and meet time-sensitive needs. Despite obstacles establishing data pipelines for non-traditional data, once in place, researchers often experienced more reliable access than to traditional data. This enabled them to use these sources while awaiting data from hospital and surveillance systems that were overwhelmed by competing demands. Medical and public health administrations appeared unprepared to share data externally, often releasing it with delays and in formats that required extensive cleaning and extraction before use. Nevertheless, participants emphasized that non-traditional data was complementary to traditional sources, rather than a replacement.

Mobility and participatory surveillance data were especially well-suited to compensate for shortcomings of traditional data streams by providing earlier signals on the spread and transmission of the virus when they were most urgently needed. Mobility data was believed to have played a crucial role in enhancing predictive and epidemiological models, assessing lockdown compliance and conducting counterfactual and forecast analyses.

“*We proposed [to policymakers] that we would analyze mobility data to predict the evolution of the pandemic. We were looking to integrate epidemiological data with mobility in order to have a model that would take into account that mobility. Mobility data helped to better predict how the pandemic would evolve in different scenarios and thus facilitate decision making based on this information.”* (P10, Research, Spain)

Participatory surveillance was able to provide syndromic data faster and capture healthy individuals who would have otherwise gone unreported. Platforms could also survey participants about their contacts, test results, severity and duration of symptoms, compliance with NPIs, vaccination status, ability to cope with confinement, perceived risk, and more recently, symptoms of long COVID.

“*The idea is that [participatory] platforms are embedded in traditional surveillance activities to capture aspects of the disease that can't be captured through traditional surveillance. Like cases of people who are not visited by doctor, or if they saw a doctor, took medicines, took days off work or school, were vaccinated previously... And during COVID, we added additional questions to get information on testing, compliance with restriction or self hygiene measures. You can capture really detailed information that other systems cannot.”* (P04, Research, Italy)

Social media was highlighted as a valuable tool for assessing sentiments surrounding vaccines and government interventions, as well as monitoring the spread of information. Researchers believed that social media could establish a feedback loop between policymakers and the public, offering a less invasive, cost-effective, and quicker alternative to a survey. Moreover, it held the potential to mitigate social desirability bias and capture more genuine expressions of opinions and attitudes.

##### 3.2.1.2 Subtheme 2: potential of non-traditional data underutilized

Despite the many benefits of non-traditional data, the consensus among participants was that they were underutilized by policymakers and public institutions, specifically public health. The use of mobility and participatory surveillance data by policymakers, for example, varied greatly and was less likely to be applied at the regional or province level where impact could have been significant. Although participatory surveillance programs have generated increased interest in their adoption as a routine surveillance activity, participants believed that far more countries and institutions could benefit than currently are. Social media data, while recognized for having potential to inform public health campaigns, made contributions that were mainly viewed as scientific findings and could be better leveraged.

Moreover, there was a shared perception that opportunities to leverage and integrate non-traditional data for public health purposes beyond the pandemic were overlooked. This was attributed to multiple factors affecting public institutions and certain research communities, including a lack of awareness, weak data infrastructure, insufficient funding for technical staff, resistance to change, and distrust of novel data sources. Participants underscored applications of mobility data for public health in particular, by capturing metrics related to access inequality, climate change, migration and displacement, air pollution, energy efficiency, housing, transportation, and sedentary behaviors.

“*Mobility data and more generally non-traditional data sources are very useful for many issues, in particular related to sustainable development goals, especially reducing inequalities. Mobility data is very important in this context because if you can easily access hospitals or education facilities or your job, this will have an impact on your everyday life and poverty level. So I think there is a lot of power and information we can still extract from this data to learn more about barriers you can encounter that has an impact on inequality.”* (P03, Research, Italy)

#### 3.2.2 Theme 2: facilitators to non-traditional data use during COVID-19 pandemic

We identified three facilitators that made it easier for participants to leverage non-traditional data during the pandemic. These included: (1) having more and faster data access; (2) collaborating across discipline and institution; and (3) pre-existing data infrastructure and institutional preparedness.

##### 3.2.2.1 Subtheme 1: expanded and rapid access to data

All participants encountered increased access to non-traditional data during the pandemic as a result of widespread interest in contributing to pandemic efforts. Though gaining access still posed challenges, many believed acquiring the data required less time or financial expense compared to non-pandemic circumstances. Participatory surveillance researchers, for example, reported achieving larger sample sizes than before due to unprecedented citizen engagement. In addition, a surge of willingness within the private sector to contribute resources resulted in the provision of anonymized, preprocessed data at little to no cost. Specifically, many telecommunication and social media companies dedicated teams to support COVID-19 data requests. In Spain, for example, the main telecommunications companies began to share anonymized mobility datasets following the state of emergency declaration.

“*During COVID some things were moving towards an ideal scientific collaborative environment. Even from companies that traditionally keep their data private were more open to collaborating... Things became easier because big companies realized they could help and contribute. Telecommunications companies and social media companies became more collaborative. There were many new opportunities in that respect.”* (P01, Research, Italy)

##### 3.2.2.2 Subtheme 2: unlikely collaborations across disciplines and institutions

Participants believed collaborations that formed during the pandemic, often between unexpected partners, were pivotal to utilization of non-traditional data. Participants, predominantly working in Spain, observed and participated in new alliances formed between governments, research institutes, universities, corporations, and citizens. Participants felt that the urgency of the crisis fostered an environment of experimentation and cooperation in places where bureaucracy, distrust, and resistance to change have historically impeded progress.

“*The difference with this [advisory group], and I think it's something we should learn, is that it's worthwhile to create a group of very different people, with very different expertise, because they enrich a lot the decision making. Working with people different from us [in the Public Health Department] has been key to better understand what was happening. And in Spain it is not usual to work with people with very different expertise.”* (P17, Public Official, Spain)

The most noteworthy collaborations were those that connected data to policy. Participants who were actively involved in a taskforce or advisory group with policymakers, whether at the regional or national level, believed non-traditional data directly informed response efforts during the pandemic. These collaborations were described as transformative, novel, and a promising model for evidence-based policy making. Participants believed the success of transferring insights from nontraditional data to policy depended on having government leadership genuinely committed to data-driven decision making and an intermediator skilled at translating complex results into policy actions.

“*In the Valencian Community there has been a very direct relationship between political authorities and technicians. We gave them almost daily information and weekly analysis of the situation. Not only [that], but they read it, studied it and asked us questions. This does not happen other places where nobody reads the reports. Here they read them and that made the work much easier because they really listened to us. This did not happen in Spain usually. In our case it has and that has been to everyone's benefit because at least from the decisions made we had been listened to.”* (P17, Public Official, Spain)

Participants viewed their experience in science-to-policy collaborations positively and were interested in participating in similar efforts again. Those from research and public health backgrounds described their involvement as motivating, and for many it marked the first time they felt their work had been listened to by government officials. For university-based researchers relationships were opened with other researchers and with policymakers in an unprecedented way, resulting in better application of non-traditional data. Moreover, some participants emphasized that efforts to collaborate with scientists from different institutions, who were similarly utilizing the data, improved the consistency, reproducibility, and harmonization of non-traditional data. Collective efforts promoted the sharing of open-access code, the creation of toolkits, as well as the adoption of best practices and privacy-preserving data use. Participants working in policy described how these collaborations also shifted norms within government structures by improving data sharing and infrastructure, increasing data-driven decision making, and bringing about new awareness to the value of non-traditional data.

“*Everything for us [in regional government] has changed. We have been able to break [down] walls. All our technological effort and collaboration to build a data dashboard for health policy decisions through COVID made it possible to continue calling on different research groups. First, in terms of government change, it was very transformative. Now everyone is much more used to sharing data for policy decisions. Second, it has empowered research. For probably one of the first times research groups can say: ‘what I recommend or say is actually being read and considered for a decision'.”* (P18, Public Official, Spain)

##### 3.2.2.3 Subtheme 3: existing infrastructure and preparedness

According to many participants, having a well-established data infrastructure and institutional preparedness significantly facilitated the effective and efficient application of non-traditional data to pandemic efforts. Systems already in place for handling non-traditional data enabled teams to swiftly pivot research to COVID-19 focused work. These included data pipelines, data use contracts, data collection platforms, and sufficient computing power. Also critical to leveraging non-traditional data was having a team equipped with relevant technical expertise and experience, along with pre-existing collaborations with data owners.

“*We were doing a lot on vaccine hesitation via social media and sentiment tracking around interventions to understand the mood of society, and gap between perceived and actual COVID risk. We were ready in the sense that we had pipelines in place for years. For participatory surveillance we were super ready because we had the staff and technical platforms in place to collect this data.”* (P07, Research, Italy)

#### 3.2.3 Theme 3: challenges to using non-traditional data use during COVID-19 pandemic

Challenges of non-traditional data use during the pandemic fell into three sub-themes: (1) minimal control over data; (2) data quality concerns; and (3) inadequate granularity for exploring inequities.

##### 3.2.3.1 Subtheme 1: minimal control over data

Excerpts regarding challenges focused on a lack of control over access, collection, and pre-processing of data, specifically mobility and social media. Researchers experienced frustration over reliance on private data owners that could abruptly modify or rescind access. Company methodology on how data was collected, aggregated or reported could also occasionally change, impacting comparisons of data across time and space. Some participants also expressed concerns over the lack of company transparency regarding methodologies used. Finally, harmonizing across diverse data sources remained an ongoing challenge, especially with mobility data where the geographic detail, technology used and methodologies employed frequently varied by source.

“*The limitations related to big data are due to the fact that they are really messy, noisy, not so specific. And most important, they are privately owned and held by companies. At the moment, they closed the faucet [of data] with Facebook, Instagram, Twitter. Basically, you don't have access to those data anymore and you don't even know how they have been collected, how they are processed, and you don't have the consent of the users to use those data to do surveillance.”* (P04, Research, Italy)

##### 3.2.3.2 Subtheme 2: data quality challenges: representativity, bias and accuracy

The issue of data quality surfaced as a frequent and burdensome challenge for participants. Obtaining data from online platforms or telecommunications companies meant that samples were not designed to be representative of the population, posing inherent risks of selection bias. Participants highlighted limitations for all types of non-traditional data included in this study; acknowledging that mobility data may miss populations with lower smartphone ownership, participatory surveillance is likely to reach individuals already committed to health, and social media data captures engaged users of platforms that appeal to specific demographic profiles. Consequently, the data are susceptible to over or under-representation based on the data collection mode and affiliated characteristics.

“*Even very simple things like demographic representivity. Who is using Snapchat or TikTok? They are totally different people from those using Twitter... The basic demographics of social media platforms are totally different. So you have no or limited control of who the author is of what you are reading. You're also passing through the black box that is the recommender system of each social media platform and you don't know how it works.”* (P06, Research, Italy)

Participants also encountered challenges extracting meaningful insights from non-traditional data due to data gaps and the absence of verification for proxy measures. Online discussions, for example, are generally limited to mainstream topics, missing sensitive topics or those affecting specific populations. Beliefs, intentions, or actions shared online could not be authenticated to determine if they reflected actual health behaviors. Evaluating genuine sentiments was particularly problematic with polarizing topics such as vaccination or mobility restrictions. Similarly, self-reported information from participatory surveillance platforms, including vaccine status, test results or healthcare visits could not be verified. Lastly, the accuracy of mobility data could be impacted by the signal strength of the mobile phone, window of error, and whether an individual used multiple devices or one device was shared by multiple people.

##### 3.2.3.3 Subtheme 3: inadequate granularity for exploring inequities

When discussing challenges, participants emphasized that social media and mobility data often lacked essential demographic and geographic details for assessing inequities. Inadequate data granularity prevented researchers from capturing disproportionate impacts of the pandemic, thereby limiting decision-makers' ability to respond to inequities. Although many acknowledged that too much detail could compromise privacy protections, the majority believed in the potential of technical solutions to mitigate this issue. Social media data rarely provided details on user characteristics, leaving researchers to infer information from profiles. Geographic detail of mobility data often was restricted to large geographic areas, preventing the analysis of smaller areas such as municipalities or neighborhoods. Most participants also noted that occasionally more detailed analyses were possible but not prioritized due to time constraints during the pandemic. However, many found this to be significantly problematic and expressed a commitment to address it.

“*[Mobility] data had very little precision, resolution, and detail to preserve the anonymity of the data. This was a problem because not having or having very few details, the data models and vizualizations have to be very generic... We could not know ages or genders. Maybe knowing that would have helped us know if someone was going to work or school or leisure activity. But we did not have details to sectorize to say if movement was higher in men or women or children or elderly.”* (P09, Research, Spain)

#### 3.2.4 Theme 4: recommendations for future use of non-traditional data

To maximize public value of the non-traditional data discussed in this study, participants focused on three recommendations: (1) strengthen data governance and infrastructure, (2) institutionalize collaborations, and (3) enhance data quality, utility, and public trust.

##### 3.2.4.1 Sub-theme 1: strengthen data governance and data infrastructure

Participants collectively advocated for enhanced data governance to address issues of data availability, usability, integrity, and security. Individuals working with mobility and social media data struggled to navigate the numerous and often conflicting priorities of stakeholders from civil society, public health agencies, and tech companies. The absence of an intermediate governing body caused fragmented efforts and data delays for those attempting to leverage non-traditional data during the pandemic. To address this, participants proposed establishing a novel institution that could improve data governance by acting as a designated data broker. This entity could be responsible for overseeing access, harmonizing diverse data sources, and establishing uniform policies related to consent, privacy, and use. Furthermore, it could manage relationships with data owners, interface with the raw data as needed, define data sharing protocols, standardize solutions for data limitations, and sustain data pipelines. Some also suggested an observatory that incorporated non-traditional data into public health surveillance and could generate routine reports, provide policy recommendations, and be activated for emergencies.

“*We are trying to crystallize some of the lessons learned from using this data. To have better data governance strategy and better public value generation we need new collaborations, institutions or type of roles. Some are arguing for an institutionalized data steward figure of companies and industries and public health agencies and research institutions so this group of people are interfaces for data use and sharing at their own institution. And you can activate this group fast in an emergency. I resonate with that because in the early phases of COVID, it took us ten days to find who at telcos to even start the discussion on data. There was no institutionalized interface.”* (P07, Research, Italy)

Participants also identified a need for sustained investment in data infrastructure to efficiently use both non-traditional and traditional data, particularly in public domains like universities, public health departments, and government agencies. Specific priorities included automated access pipelines, secure databases, personnel for data processing, and analytics software. Unique challenges arose when working with public health and hospital systems, revealing issues such as data silos, insufficient data infrastructure, and lack of data sharing procedures. Despite the initial success in setting up numerous data pipelines during the pandemic, many believed much of this infrastructure was now underutilized and unmaintained due to other priorities. As a result, participants stressed the critical importance of investing in existing data pipelines, building new ones as necessary, and institutionalizing standards for usage.

“*Beautiful pipelines have been created, but now they have to be maintained. We need the companies who, rightfully so, are telling us 'this is not our job at this moment, we cannot do that forever.' And this can be done only if we create brokers able to work with the companies and academic world and agencies like CDC to create a system that provides the resources and incentive to the business world. That's essential and if we don't do that, we will lose everything we have done so far. And the next time we will have to begin from scratch all over again.”* (P08, Research, Italy)

##### 3.2.4.2 Sub-theme 2: institutionalize collaborations across stakeholders

In excerpts on recommendations participants emphasized the importance of institutionalizing diverse collaborations, especially between researchers and policymakers, to effectively utilize non-traditional data for public health beyond the pandemic. Those engaged in these collaborations believed they led to improved data access, scientific results, and data-driven policy. However, participants noted that most were designed to be temporary and were unsustainable due to heavy reliance on volunteer efforts. Despite their perceived ongoing value, advisory boards linking scientific and policy sectors were dissolved amidst the decline of COVID-19 cases. Public-private partnerships that were strengthened during the pandemic, were also observed to have waned as companies limit data sharing.

“*I believe that it has made it very clear that in order to solve certain things we need a team. That no one by themselves with their own techniques is capable of solving a very big problem. So, the fact of being able to collaborate and establish these networks I think is something that the whole issue of COVID has made clear that it is necessary—funding to promote public-private collaborations.”* (P12, Research, Spain)

Participants urged the allocation of resources and incentives to engage research groups, government entities, public health agencies, companies, and other stakeholders in collaborative efforts. Participants emphasized the value of a dedicated staff member within government who could serve as the link between researchers and decision makers. Many participants, drawing on their experiences during the pandemic, highlighted the impact this role had on facilitating evidence-based policy making. Researchers from Spanish universities also believed that the culture within the traditional academic environment discouraged and did not value their staff participating in external collaboration with other universities or government agencies. In response, they proposed incorporating incentives into promotion and tenure policy to foster continued collaboration. Lastly, participants from the public sector (policy and public health) emphasized that improving technical capacity of their workforce and data infrastructure would facilitate more effective collaborations between science and public policy.

“*The only way to take advantage of this new data science [at the public health department] is to have [trained] people to allow us to connect with research teams... In order for research to be transferred to daily lives, we need a bridge that connect us. And that is these professionals, because for us all of this is very new. We are making a mistake if we don't start connecting with teams using artificial intelligence [and non-traditional data]. Because it is very clear to me that it is one of the key tools and we have to innovate. Artificial intelligence, using sewage water to study things... This taskforce was the first time I'd worked with very different people and it has opened my mind a lot.”* (P17, Public Official, Spain)

##### 3.2.4.3 Sub-theme 3: enhance non-traditional data quality, utility, and public trust

Participants stressed the need to address common concerns related to non-traditional data. Many recognized that during the COVID-19 pandemic the urgency for immediate results often took precedence over the time required to address data issues of representativity, bias, and disaggregation. However, participants emphasized the importance of developing new or refining existing analytical techniques and methodologies that enhance the quality of metrics produced with non-traditional data. This was especially relevant to the study of inequities, which was neglected during the pandemic due to the urgent demand for *any* data.

“*This is why we do science on this, because those data were not collected in the classic double blind or with full representation of the population. These are massive data which have the statistical power of millions of individuals, but are not stratified in the proper way and are biased. This is why there is scientific work on this. And in peace time, we try to improve the quality of those data. For instance, we got mobile data for millions of users, but then restrict it to a representative panel of users. And just that process might take months, if not years.”* (P08, Research, Italy)

Several researchers were focused on how to incorporate or gain access to socio-demographic factors in order to identify inequities using non-traditional data. However, they faced challenges with obtaining access and ensuring data was still aggregated at a level that safeguards privacy. Some participants who made progress in this area during the pandemic, expressed disappointment at the lack of funding and prioritization for these methods. Alongside technical concerns, many emphasized the importance of addressing concerns over privacy and user consent. Researchers saw an opportunity to improve communication about the value and safety of non-traditional to generate awareness and trust among the public. Finally, some advocated for greater citizen empowerment over their digital data and the decision to share it.

## 4 Discussion and implications

Based on our qualitative analysis, we draw several implications regarding the use of non-traditional data, specifically mobility, social media, and participatory surveillance, in the context of a pandemic.

### 4.1 The value of non-traditional data

The findings of this study shed light on ways some non-traditional data sources made unique contributions throughout the pandemic. This is supported by recent publications that document the untapped potential and advantages of non-traditional data for public health surveillance, especially their high-frequency, high-volume, and minimal effort data collection methods (2, 44, 45). Participants described the pandemic as a catalyst for the increased acceptance and appreciation of non-traditional data, especially within the government and traditional public health sector. Participants saw opportunities to incorporate them into data systems and regular operations of public departments to offer new insights and strengthen decision making. In our case study, participants successfully leveraged mobile device and participatory surveillance data to inform policy making during the pandemic. Participants who analyzed social media data could not confirm that a direct link to policy was made. However, recent literature has documented their ability to offer insights into public reactions to interventions and attitudes about vaccines (28, 46, 47). There was a shared perception that opportunities to leverage and institutionalize these non-traditional data sources for future use in the public domain were being overlooked, particularly in public health. This was attributed to various factors, including a lack of awareness, weak data infrastructure, insufficient funding for technical staff, resistance to change, and distrust of the data within certain public sector and research communities. Until these challenges are addressed, the potential for non-traditional data to inform and support policymaking will not be realized.

### 4.2 The need for sustained efforts

There was consensus among participants that many initiatives to use non-traditional data had not been sustained despite holding significant value beyond the pandemic. While efforts to apply non-traditional data to socioeconomic and recovery strategies are ongoing (48), many others have stalled. Participants believed there was insufficient political will to invest in non-traditional data use, especially in the public sector. Participants emphasized the importance of sustaining collaborations, particularly those bridging science and policy, and investing in data infrastructure. These findings build upon recent research (49) that identified similar opportunities to better utilize digital data through data science training opportunities for public health experts, establishing public and private partnerships, and addressing biases and privacy concerns. They are also supported by historic publications, including discussions nearly a decade prior on the potential of public health informatics to improve population health. Recommendations documented by Edmunds et al. (50) highlighted the need to strengthen intersectoral collaborations, data infrastructure, public health workforce capacity and communication with elected officials. Our study found that public health and other government agencies struggled to acquire, process, and share the overwhelming amount of data required to quickly respond to the pandemic. In the case of Spain, this has also been documented elsewhere by Royo (51), who noted a lack of available and reliable data during the pandemic, which undermined public confidence and trust. While this often referred to traditional data sources, our conclusion is that these areas need further investment in order for public health and other public agencies to fully leverage all relevant sources of data. This aligns with recent literature and reports that document the need to develop data infrastructure, pipelines, and evidence-based culture within government agencies (52, 53) as well as data collaboratives to leverage private-sector data (54).

### 4.3 Data governance should be a priority

A prevailing recommendation that emerged from this study was for a coordinated focus on improved governance of non-traditional data. Data governance has been raised as a priority by a wide range of stakeholders invested in maximizing the public value of data, including the United Nations (World Data Forum), World Health Organization (Health Data Governance Summit), The European Commission (High-level Expert Group on Business to Government Data Sharing), The Lancet, Financial Times (Governing Health Futures 2030 Report) and Transform Health, among many others. Although the adoption of the General Data Protection Regulation (GDPR)^1^ in 2016 by the European Union aimed to address some of the needs for data governance, shortcomings have been identified with it's ability to regulate the expansive availability of digital health data (55).

Initially, data governance efforts concentrated primarily on traditional health data, but has since broadened in response to the growing reuse of data generated from digital technologies for health-related purposes (56–58). Additional efforts to create data governance frameworks have taken place by the Development Data Partnership, the OECD, the Council of Europe, and the World Health Organization, however gaps remain. A challenge with the multitude of data governance efforts is that frameworks often conflict or overlap, while not being legally binding and therefore difficult to enforce (59).

Many study participants specifically identified a need for common practices around data access and management, as well as guidance on ethical concerns regarding data bias, privacy safeguards, and consent. Recent attempts to address this within Europe can be found in the European Data Governance Act^2^ from 2020 and its proposed complement with the European Data Act^3^ from 2023, the European Digital Services Act^4^ from 2022 and the upcoming European AI Act. While participants acknowledged the progress of these steps, some felt the language were too idealistic, lacked specificity, and were challenging to operationalize. Commentary on these issues includes calls for more participatory approaches, inclusion of equity and human rights, and representation from groups especially impacted, such as marginalized communities (60, 61). Additionally, several conceptual frameworks have been proposed, including ones rooted in data solidarity and data justice (62), principles of equity, individuals rights (59), and antiracism (63).

### 4.4 Key data limitations need to be addressed

A principal finding that emerged throughout this study was that non-traditional data, while valuable, also has complex challenges regarding bias, representation, generalizability, and access. The qualitative interviews provide important insights on concerns regarding data limitations, which centered on the lack of control over data collection methodology, as well as availability of geographic and demographic detail. These findings build upon recent studies that highlight the need to strengthen privacy guardrails (64), address user consent (65), and reduce data bias (66, 67) of non-traditional sources. The importance of collecting socioeconomic characteristic data that identify high-risk populations has been widely recognized in the field of public health. Although advocated at the beginning of the pandemic, as seen in the work of Khalatbari-Soltani et al. (68), it continues to be challenge, especially for non-traditional data sources. Participants felt there was an ethical dilemma between how to sufficiently protect data privacy and have enough detail to identify disproportionate impacts across populations. The disproportional impact COVID-19 had on high-risk populations demonstrates the critical need to have data that can identify health inequities and improve policy response.

### 4.5 Future research needed

This study revealed several opportunities for future research. First, our results do not quantify the impact that non-traditional data use had on policy or health outcomes during the pandemic. We therefore emphasize the need to expand the evidence base on the effectiveness of public health initiatives that used non-traditional data. This study highlighted the need for public agencies to enhance data infrastructure and bolster technical workforce capacity to better utilize both non-traditional and traditional data. While this captured perspectives on public health departments, it also included references to healthcare and policy-focused agencies. To better understand the public health context, we believe future research should further explore and assess these needs specifically within public health departments. Lastly, additional research is necessary to identify and document strategies for reducing the biases of non-traditional data and better leveraging it to advance health equity work while ensuring privacy protection and appropriate consent.

## 5 Limitations

There are several limitations to this study. First, the recruitment strategy used relied on the investigators' knowledge and snowball sampling, which may have resulted in the absence of important perspectives in the research sample. Additionally, participants worked primarily in Italy and Spain during the pandemic and though their work was not limited to these areas, most of their experiences were specific to the context of those countries. We also acknowledge an unbalanced sample by gender with twice as many male than female participants; by country with slightly more participants working in Spain than in Italy; and by data focus with more knowledge of mobility and participatory surveillance than of social media or internet activity. We would have liked to include more individuals from the public sector, specifically public health and policy, however encountered challenges with recruitment due to candidates' busy schedules. Second, one of the lead investigators was engaged in non-traditional data use through a taskforce during the pandemic and had working connections to multiple interviewees. This may have biased their perceptions of findings based on their individual experiences, attitudes, and beliefs. To minimize this influence, the investigator voluntarily refrained from leading data collection or analysis activities, and instead reviewed the results alongside access to de-identified and anonymized primary data.

## 6 Conclusion

Our study provides important insights on the utilization of non-traditional data to combat a global pandemic, with specific exploration of their applied value, facilitators and challenges encountered during use, and future recommendations. Participants described key facilitators that made using non-traditional data more effective and efficient during the pandemic. These included increased collaborations, expanded data access, and pre-existing technical capacity and infrastructure. Despite this, the end of the pandemic led to a loss of many of these facilitators, namely collaborations and data access. Common challenges encountered by participants related to lack of control over the data source and data inadequacies, particularly when it came to the granularity of the data. Recommendations identified in this study build upon existing literature and include (1) strengthening access and standards of use through a data governance frameworks and data brokers; (2) investing in diverse and sustained collaborations; and (3) prioritizing technical solutions to concerns in order to increase data utility and public trust. Research institutions, multilateral organizations, governments, and public health authorities may be able to draw lessons from these findings when considering how to strengthen the utility of non-traditional data for public health and beyond.

## Data availability statement

The datasets presented in this article are not readily available because data collected for this study will not be made available to others given confidentiality of the interviews and ethical and institutional regulations. The summary of themes and corresponding quotes from interviewees are included in the Supplementary material. Further inquiries can be directed to the corresponding author. Requests to access the datasets should be directed to kabolt@kingcounty.gov.

## Ethics statement

The studies involving humans were approved by Ethics Committee at University of Alicante. The studies were conducted in accordance with the local legislation and institutional requirements. The ethics committee/institutional review board waived the requirement of written informed consent for participation from the participants or the participants' legal guardians/next of kin because consent was provided electronically (signed) through Google Forms prior to the interview and through verbal agreement at the beginning of the interview. Questions were deemed non-sensitive in nature and minimal risk to participants.

## Author contributions

KB: Conceptualization, Data curation, Formal analysis, Funding acquisition, Investigation, Methodology, Supervision, Writing—original draft, Writing—review & editing. DG-G: Conceptualization, Methodology, Supervision, Validation, Writing—review & editing. NO: Conceptualization, Methodology, Resources, Supervision, Validation, Writing—review & editing.

## Footnotes

^1^ https://eur-lex.europa.eu/eli/reg/2016/679/oj

^2^ https://eur-lex.europa.eu/legal-content/EN/TXT/?uri=CELEX%3A32022R0868

^3^ https://eur-lex.europa.eu/legal-content/EN/TXT/?uri=COM%3A2022%3A68%3AFIN

^4^ https://eur-lex.europa.eu/legal-content/EN/TXT/?uri=CELEX%3A32022R2065&qid=1666857835014
