# Supplementary material for: Unconventional data, unprecedented insights: leveraging non-traditional data during a pandemic
Source: Front Public Health. 2024 Mar 7;12:1350743. doi: 10.3389/fpubh.2024.1350743 (PMC10986850; doi:10.3389/fpubh.2024.1350743)
Supplement: Supplementary file 1 [file Data_Sheet_1.PDF]

**Introductions and Opener**	
1.	Can you state your name, position title and how long you have been in that role?
**Utility of non-traditional data use during COVID-19 Pandemic**	
2.	For the [insert non-traditional data type] data, can you describe the way you have been using this data during the pandemic?<br>• How was it useful?<br>• What were the strengths of using this data?
3.	How have you seen this data be used in [Spain / Italy] during the pandemic compared to:<br>• Previous approaches; other approaches across Spain / Italy; in the EU or globally
**Facilitators of using non-traditional data**	
4.	What helped facilitate its use during the pandemic?<br>• Could you describe what made the process easier?
**Challenges of using non-traditional data**	
5.	What challenges were encountered when using this data during the pandemic and how were they addressed?<br>• Could you describe what made the process more difficult?<br>• What could be improved?
**Data privacy and equity**	
6.	How did privacy protections impact this work?<br>• What were the privacy risks?<br>• How was data privacy protected and risks mitigated?<br>• What challenges emerged in protecting privacy throughout the work
7.	How did the use of [insert non-traditional data type] consider equity in the approach?<br>• Who did the data capture and who could it have missed?<br>• What data would you have liked to have?
**Successes and impact**	
8.	What do you see as the most successful outcome(s) of your use of [insert non-traditional data type] data during the pandemic so far?<br>• What was critical to success?
9.	How well was it used to inform policy decisions?<br>• What would have made it easier to inform policy?
**Future recommendations**	
10.	In your view, what is the most significant impact that [insert non-traditional data type] data can have in the future?<br>• How well do you think it is currently being utilized or recognized as a useful data source? (By public health, by the public sector, etc.)<br>• Do you think the value of this data is understood?

11.	How could the work with [insert non-traditional data type] data during the pandemic be applied to future pandemics or public health emergencies?<br>• How could it be applied to non-emergency situations?<br>• What lessons learned are important to share for future use of these data sources?
12.	What recommendations do you have to better use this data for public health?<br>• For social good in general?<br>• What changes need to happen to do that?
