# Supplementary material for: Unconventional data, unprecedented insights: leveraging non-traditional data during a pandemic
Source: Front Public Health. 2024 Mar 7;12:1350743. doi: 10.3389/fpubh.2024.1350743 (PMC10986850; doi:10.3389/fpubh.2024.1350743)
Supplement: Supplementary file 2 [file Data_Sheet_2.PDF]

Summary of Findings by Topic Guide Questions	
**Utility of non-traditional data use during COVID-19 Pandemic**	
Question	For the [insert non-traditional data type] data, can you describe the way you have been using this data during the pandemic?
Findings	**Applications of Non-Traditional Data:**<br>• Mobility and participatory surveillance data facilitated early signals on virus spread, enabled evaluation of mobility restrictions and other nonpharmaceutical interventions, and compensated for traditional data limitation<br>• Social media data provided insights into public opinion and emotional states by assessing sentiments, which offered a cost-effective alternative to surveys and fostered a feedback loop between policymakers and the public<br>**Unique Advantages of Non-Traditional Data:**<br>• Addressed critical gaps and provided timely insights<br>• Initially more reliable access over traditional sources
Question	How have you seen this data be used in [Spain / Italy] during the pandemic compared to previous approaches; other approaches across Spain / Italy; in the EU or globally, etc?
Findings	**Limited Global Comparisons:**<br>• Majority of participants did not directly compare across countries or globally<br>• Many felt unequipped with the expertise for such comparisons<br>**Institutional Openness and Collaboration:**<br>• Increase in institutional openness to new ideas and collaboration, motivated by the urgency of the pandemic<br>• Emergence of new efforts to use non-traditional data for policy and decision-making<br>**Variation in Use:**<br>• Mobility and participatory surveillance data use varied across the country, with less application at regional levels where significant impact was possible
**Facilitators of using non-traditional data**	
Question	What helped facilitate its use during the pandemic?
Findings	**Expanded and Rapid Access to Data:**<br>• Increased willingness from the private sector to offer support and data access<br>• Anonymized, preprocessed data provided at little to no cost<br>**Collaborations Across Disciplines and Institutions:**<br>• New alliances formed between governments, research institutes, universities, corporations, and citizens<br>• Collaborations connecting data to policy viewed as particularly novel and transformative<br>**Dependency on Government Leadership:**

	• Success of collaborations connecting data to policy dependent on government leadership committed to data-driven decision-making<br>**Existing Infrastructure and Institutional Preparedness:**<br>• Well-established data infrastructure and institutional preparedness<br>• Swift pivot to COVID-19-focused work facilitated by data pipelines, contracts, collection platforms, computing power, and technical expertise
**Challenges of using non-traditional data**	
Question	What challenges were encountered when using this data during the pandemic and how were they addressed?
Findings	**Minimal Control Over Data:**<br>• Frustrations over reliance on private data owners<br>• Lack of transparency or full knowledge of company methodologies<br>**Data Quality Challenges:**<br>• Representativity issues, bias, and accuracy concerns<br>• Non-representative samples from online platforms or telecommunications companies<br>• Inherent risks of selection bias<br>**Challenges in Evaluating Sentiments:**<br>• Data gaps and absence of verification for proxy measures<br>• Difficulty in evaluating genuine sentiments on polarizing topics<br>**Inadequate Granularity for Exploring Inequities:**<br>• Social media and mobility data lacked essential demographic and geographic details<br>• Hindered researchers' ability to capture disproportionate impacts of the pandemic<br>• Limited decision-makers' ability to respond to inequities
**Data privacy and equity**	
Question	How did privacy protections impact this work?
Findings	**Challenges of Privacy:**<br>• Privacy protections made it challenging to share data with other researchers<br>• Ethical tension existed between protecting data privacy and having enough geographic and demographic detail to identify disproportionate impacts across populations<br>• Belief in the potential of technical solutions to address privacy issues<br>**Need for Citizen Empowerment:**<br>• Some advocated for greater citizen empowerment over their digital data and user consent
Question	How did the use of [insert non-traditional data type] consider equity in the approach?
Findings	**Disproportional Impact on High-Risk Populations:**

<table>
<tr><td></td><td>Participants felt equity was not an immediate focus at the onset of the pandemic as the prevailing confusion and urgency to gather any information on virus containment took precedence
Participants recognized that the disproportional impact of COVID-19 on high-risk populations highlighted the critical need for data to identify health inequities and improve policy response
Inadequate Detail in Data:
Participants highlighted challenges, especially in social media and mobility data, which lacked essential demographic and geographic details for assessing inequities</td></tr>
<tr><td colspan="2">Success and impact</td></tr>
<tr><td>Question</td><td>What do you see as the most successful outcome(s) of your use of [insert nontraditional data type] data during the pandemic so far?</td></tr>
<tr><td>Findings</td><td>Improved Data-Driven Policy Response
Participants believed the most successful outcomes were that non-traditional data, such as mobility, social media, and participatory surveillance, was able to directly inform policy decisions and improve response efforts
Policymakers and researchers established collaborations, improved data infrastructure and pipelines, and strengthened data-driven decision making
Acceptance and Understanding of Utility
The pandemic acted as a catalyst for increased acceptance and appreciation of non-traditional data in government and traditional public health sectors
Opportunities were identified to incorporate these data into regular operations of public departments, offering new insights and strengthening decision-making</td></tr>
<tr><td>Question</td><td>How well was it used to inform policy decisions?</td></tr>
<tr><td>Findings</td><td>Direct Impact Non-traditional Data to Policy:
Non-traditional data, especially mobility and participatory surveillance improved accuracy of forecasting and predictions of COVID-19 rates and spread
Participatory surveillance provided much earlier signals of outbreaks, a better understanding of symptoms, insights on behaviors, and informed resource allocations
Level of Policy Impact Varied
Impact on policy was limited by delays in access, political resistance, and levels of awareness or trust in non-traditional data sources
Benefits of non-traditional data went underutilized in part due to lack of awareness, preparedness and inadequate data infrastructure</td></tr>
<tr><td colspan="2">Future Recommendations</td></tr>
<tr><td>Question</td><td>In your view, what is the most significant impact that [insert non-traditional data type] data can have in the future?</td></tr>
<tr><td>Findings</td><td>Maximize Public Value of Non-traditional Data</td></tr>
</table>

<table>
<tr><td></td><td>

- Participants felt non-traditional data was generally underutilized in the public sector and felt that they could be better leveraged or use could be expanded
- Mobility data in particular was highlighted for applications related to access inequality, climate change, migration and displacement, air pollution, energy efficiency, housing, transportation, and sedentary behaviors

Sustained Collaborative Efforts Needed:

- Participants felt there was a lack of sustained public initiatives or investment in the use of non-traditional data beyond the pandemic
- Participants emphasized the importance of sustaining science-policy collaborations and investing in data infrastructure (including automated access pipelines, secure databases, personnel for data processing, and analytics software)

</td></tr>
<tr><td>Question</td><td>How could the work with [insert non-traditional data type] data during the pandemic be applied to future pandemics or public health emergencies?</td></tr>
<tr><td>Findings</td><td>

Data Governance as a Priority:

- In order to better utilize non-traditional data in the future, participants highlighted the critical need for improved and coordinated focus on the governance of non-traditional data
- Some proposed the establishment of a novel institution, acting as a designated data broker to oversee access, privacy, harmonize diverse data sources, and managing relationships with data owners

Enhance Non-Traditional Data Quality, Utility, and Public Trust:

- Recognition of the need to address common concerns including issues of bias, representation, generalizability, demographic details, user consent, privacy guardrails, and access
- Researchers saw an opportunity to build public awareness and trust by improving communication about the value and safety of non-traditional data

Ongoing Research Opportunities:

- The study highlighted opportunities to quantify the impact of non-traditional data on policy and health outcomes
- Emphasis on developing or refining analytical techniques and methodologies to enhance the quality and protection of metrics produced with non-traditional data

</td></tr>
</table>
